# Sex disparities in psychosocial distress among medical students with indirect exposure to the 2023 Kahramanmaras earthquakes

**DOI:** 10.1080/16549716.2026.2698916

**Published:** 2026-07-13

**Authors:** Kadir Uludag, Fatih Kara, Hongxing Wang

**Affiliations:** a Department of Neurology, Capital Medical University, Xuanwu Hospital, Beijing, China; b School of Medicine, Kars Kafkas University, Kars, Turkey

**Keywords:** Anxiety, earthquake psychology, earthquake risk factors, post-disaster health, sex differences

## Abstract

**Background:**

The psychological impact of disasters extends beyond directly exposed populations to those indirectly affected through close social networks. Sex is a critical factor of post-trauma outcomes, yet its role in shaping the mental health of indirectly exposed individuals remains underexplored.

**Objective:**

This study investigates sex-based disparities in psychosocial distress among a cohort remotely exposed to a major earthquake.

**Methods:**

Approximately 3 months after the 2023 Kahramanmaras earthquakes, 129 Turkish medical students with indirect exposure (e.g. via affected relatives or close social circles) took part in this cross-sectional study, completing a structured anxiety survey and a self-report form. The self-report form assessed academic stress, health behaviors (smoking, alcohol use, exercise, and nutrition status), and lifestyle factors.

**Results:**

After Bonferroni correction, no variable remained statistically significant. However, using the Benjamini–Hochberg false discovery rate procedure, peer bullying was associated with sex (χ^2^(3) = 13.581, p_FDR = .046, Cramer’s V = 0.324). All other chi-square tests were nonsignificant under both correction methods.

**Conclusion:**

After rigorous correction, sex differences were largely attenuated. Peer bullying showed a possible association under FDR, but not under Bonferroni, suggesting the need for larger confirmatory studies.

## Paper Context

- **Main findings:** After Benjamini–Hochberg correction (but not Bonferroni), peer bullying was associated with sex among indirectly exposed medical students; all other variables showed no robust sex differences under either correction.
- **Added knowledge:** This study demonstrates that indirect disaster exposure through social networks can produce measurable psychological distress, but sex-specific patterns are subtle and largely attenuated after multiplicity adjustment. The most reliable sex-related marker was peer bullying, though this finding did not survive the most stringent (Bonferroni) correction.
- **Global health impact for policy and action:** Post-disaster mental health frameworks should extend screening and support to remotely affected healthcare trainees. While sex-sensitive approaches remain important, they should prioritize interpersonal stressors such as peer bullying and be applied cautiously, as many initially observed sex disparities may not be robust; larger confirmatory studies are needed to guide resource allocation and targeted interventions.

## Background

Major earthquakes are abrupt natural calamities that cause not only massive physical destruction but also profound psychological distress among affected populations [1]. On 6 February 2023, Turkey experienced two devastating earthquakes – magnitudes 7.7 and 7.6—centered in Kahramanmaras [2]. Known as the ‘disaster of the century,’ these quakes devastated an area of 108,812 km^2^ across 11 provinces, leading to tens of thousands of deaths, injuries, and widespread displacement [2]. The psychosocial consequences of such events are well documented: survivors face difficulties accessing resources, lack of information, and elevated rates of anxiety, depression, and post-traumatic stress disorder (PTSD) [1,3–5].

Importantly, the psychological impact of disasters is not limited to directly exposed individuals. Indirect exposure – through close social ties with victims, media consumption, or disruption of community networks – can also trigger significant distress. For every directly affected person, many more experience secondary trauma. In the context of the 2023 Kahramanmaras earthquakes, medical students who were not physically present in the disaster zone but had family members or close friends who were affected constitute a particularly relevant indirectly exposed group. Their families represent a critical resource for emotional security and social support. According to the conservation of resources theory, the threat of resource loss – even without direct physical harm – can initiate a stress response and a resource loss spiral [5]. For these students, fear for family safety, uncertainty about the future, and disruption of daily routines (e.g. medical training) may trigger sustained psychological distress.

A robust body of literature indicates that sex is a key moderator of post-disaster mental health. Across diverse disaster settings, females consistently report higher levels of depression, and PTSD symptoms [5,6]. Evolutionary theory offers a foundational explanation: sex-differentiated adaptive pressures – females as primary caregivers evolved heightened vigilance toward threats to social networks and offspring, whereas males evolved action-oriented, risk-taking coping strategies [7,8]. In the aftermath of the 2023 earthquakes, multiple studies have documented sex-based disparities. For instance, women faced greater housing and safety problems [9,10] and experienced increased menstrual irregularities linked to post-traumatic stress [11,12]. Conversely, male survivors showed higher rates of smoking and alcohol use as coping behaviors [8]. Importantly, existing research has focused almost exclusively on directly exposed populations, leaving a critical gap regarding indirectly exposed groups.

Medical students represent a unique and understudied indirectly exposed population. They are simultaneously young adults vulnerable to post-traumatic distress and future healthcare providers essential for community resilience. Their ongoing academic and professional training may be disrupted by indirect trauma, yet no study to date has systematically examined sex-based psychosocial disparities among medical students indirectly affected by the Kahramanmaras earthquakes. Understanding this gap is urgent: if sex-specific patterns of distress and coping exist even without direct exposure, disaster mental health frameworks must expand to include targeted, sex-sensitive screening and support for healthcare trainees.

In Turkey, women were already in a vulnerable position due to pre-existing inequalities, and this vulnerability was further intensified [13]. The results of a previous study show that in the aftermath of the earthquakes, women went on carrying out their responsibilities in society, as well as in childcare and family care. The findings imply that during this time, women encountered issues with housing and safety [11]. From a sex perspective, these factors may disproportionately impact women, who are often more vulnerable to economic instability and psychological distress in post-disaster contexts. However, emerging research also highlights the role of collective agency: Erler [14] found that women’s solidarity and collective action played a crucial beneficial role in enhancing community resilience and recovery following the Kahramanmaras earthquakes. This suggests that while women face disproportionate risks, their active participation in mutual support networks may serve as a protective factor. Moreover, by identifying sex-based differences in this mechanism, the study underscored the necessity of developing post-disaster mental health interventions that are tailored to address the diverse needs of various demographic groups [15]. In another study, the research findings indicate that participants’ trauma levels are affected by the following factors: being young, female, or single; having a low income; having a pre-earthquake diagnosis of a mental illness; having been trapped under debris; experiencing the injury or death of a family member; witnessing building collapses or someone being severely injured; having one’s house destroyed or damaged; residing in temporary housing after the earthquake; losing a significant amount of property or money; and relocating to another city [16]. Moreover, earthquakes can severely affect women’s well-being, with ripple effects extending to the entire family [17,18]. Other findings indicate that women predominantly adopted protective actions and displayed people-focused conduct [19].

Therefore, this study aims to address this gap by investigating sex-based disparities in psychosocial distress among medical students with indirect exposure to the 2023 Kahramanmaras earthquakes (i.e. through affected family members). The specific objectives are as follows:

1. To quantitatively assess and compare the psychosocial impact – focusing on anxiety, academic stress, and related behavioral factors – between male and female medical students who experienced remote exposure.
2. To identify and analyze the specific behavioral and lifestyle factors (including substance use such as smoking, exercise, and nutrition status) that contribute differentially to post-disaster distress patterns across sexes within this indirectly affected cohort.

## Methods

### Participants

A total of 129 medical students, indirectly affected by the Kahramanmaras earthquake through their social networks (including family, friends, or community ties), were recruited via social media platforms, including Facebook and university official WhatsApp groups (Figure 2). All participants were studying in the same city. Among them, 35 participants (27.1%) reported having family members directly affected by the earthquake. Interested individuals voluntarily clicked the link and completed the online survey after providing electronic informed consent.

At Kars Kafkas University, where the total number of medical students was approximately 520, the final sample represented 24.7% of that population.

The remaining 94 participants (72.9%) had no direct family involvement but experienced indirect exposure through close relationships with affected individuals (e.g. friends and classmates), sustained exposure to intense national and social media coverage, and disruption of academic and professional networks in the post-disaster period. The acquisition of data occurred during a period of less than 1 month, between May and June 2023, approximately 3 months after the earthquake. All participants were Turkish citizens aged 18 years or older. The study was approved by the Kars Kafkas University’s ethics committee (Approval ID: 26 April 2023/07), and all participants provided electronic informed consent. Participation was entirely voluntary. Participants who did not provide informed consent were unable to complete the study and were therefore excluded from the analysis.

**Figure 1.**
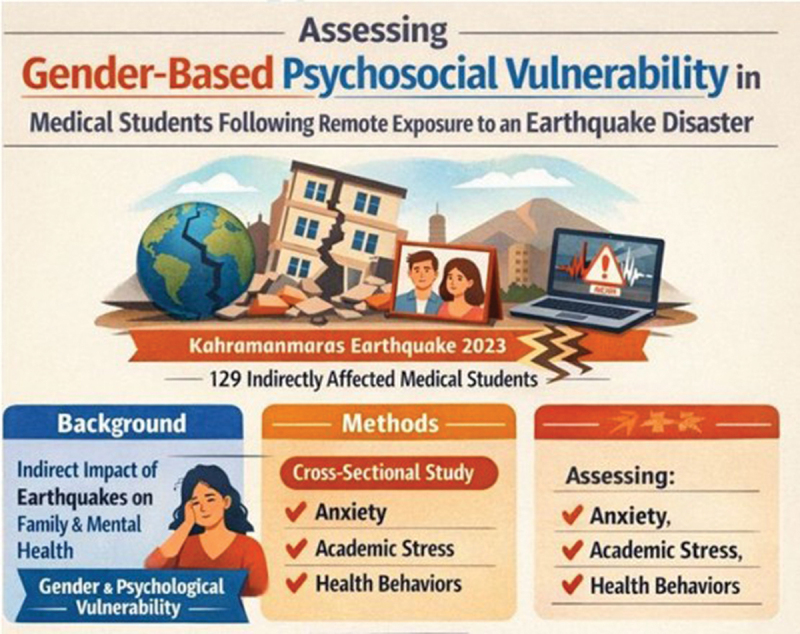
Design and key findings of the study. Originality and accuracy of content has been confirmed by the authors. The AI tool ChatGPT (version GPT-4) was used. The authors have checked the terms of use for the specific AI tool used and therefore confirm suitability for publication. The authors have the right to publish the images generated, and have obtained any permissions where required. The authors take full responsibility for the integrity of the whole content, including accuracy of references.

The final sample comprised 87 females (67.4%) and 42 males (32.6%), with 27.1% reporting that their families had been affected by the earthquake. Table 1 summarizes previous studies on sex and earthquake exposure related to Kahramanmaras. Figure 1 provides overall information related to our work. Anxiety levels were assessed using the Turkish adaptation of the Beck Anxiety Scale [20]. Scores were classified as follows: 0–7 minimal, 8–15 mild, 16–25 moderate, and 26–63 severe anxiety, consistent with the scoring guidelines for the Turkish version of the scale.

**Table 1.** Sex-related impacts of the 2023 Kahramanmaras earthquakes.

Ref.	Authors (Year)	Goal of Study	Main Results
1	Özer et al. (2025) [3]	To examine the health-related worries and experiences of women who relocated after the earthquake.	Displaced women endured a range of difficulties, with pronounced challenges in accessing healthcare, mental health support, and social services in their new locations.
2	Altuntaş et al. (2023) [4]	To explore the experiences and risk-reduction suggestions of nurses working in the affected areas.	Nurses struggled with personal psychological issues and faced systemic managerial and organizational challenges in providing care.
3	Ural et al. (2024) [9]	To gain insight into women’s experiences and health-related needs after the earthquake.	Women encountered significant housing and safety problems in the aftermath.
4	Gümüşsoy et al. (2025) [10]	To explore sex-specific challenges faced by female prehospital emergency health workers deployed to disaster zones.	Female personnel faced significant difficulties related to accommodation, and privacy in the post-disaster work environment.
5	Güzel Inal & Timur Taşhan (2025) [12]	To investigate the impact of the earthquake on women’s menstrual cycles.	The disaster and its associated stress had a measurable adverse impact on menstrual cycle regularity and symptoms.
6	Erler (2024) [14]	To explore how women’s post-earthquake experiences are shaped by sex and the role of solidarity.	Women’s solidarity and collective action played a crucial beneficial role in enhancing community resilience and recovery.
7	Kaplan et al. (2024) [17]	To examine the psychological effects of the Kahramanmaras earthquake on women.	Earthquakes exert a notable negative impact on women’s mental health and wellbeing, which affects the welfare of the entire family.

Note: Ref, Reference number.

### Statistical analysis

Statistical analyses were performed using Jupyter Notebook version 7.0.8. No missing values were identified. All variables were coded categorically as presented in Table 2. Binary variables (e.g. smoking and exercise) were coded as 1 for presence and 0 for absence. To examine associations between categorical variables, Chi-square (χ^2^) tests of independence were performed. The results of these tests, including the comparison of earthquake exposure status across demographic and psychosocial factors, are presented (Table 2). Furthermore, to investigate the relationship between sex (the primary grouping variable) and all other categorical parameters, a series of Chi-square tests were conducted. The results, including test statistics, *p*-values, and effect sizes (Cramer’s V), are summarized in Table 3. Although some expected cell frequencies were below 5 (minimum = 1.6), the chi-square test may still be valid as no expected count was below 1. The Benjamini–Hochberg procedure (α = 0.05) identified peer bullying as significant (p_FDR = .046), whereas the more conservative Bonferroni correction did not (raw *p* = .004 > .0038). All other variables were nonsignificant under both methods. The 13 categorical predictors entered into the logistic regression model (Table 4) correspond to the self-report items and coding scheme detailed in Table 5.

**Table 2.** Comparison of variables by earthquake exposure status.

	Affected through family (n = 35)	No direct family exposure (n = 94)	Asymp. Sig. (p-value)
**Academic Achievement**			.141
Successful	20 (57.14%)	54 (57.44%)	
Non-successful	7 (20.0%)	8 (8.51%)	
undecided	8 (22.85%)	32 (34.04%)	
**Occupational Satisfaction**			.014
Satisfied	14 (40.0%)	64 (68.08%)	
Unsatisfied	10 (28.57%)	13 (13.83%)	
Undecided	11 (31.4%)	17 (18.08%)	
**Anxiety**			.763
Anxious	22 (62.85%)	63 (67.02%)	
Non-anxious	9 (25.71%)	24 (25.53%)	
Undecided	4 (11.42%)	7 (7.44%)	
**Economic Status**			.068
Very good	0 (0%)	2 (2.13%)	
Good	7 (20.0%)	34 (36.17%)	
Normal	19 (54.28%)	50 (53.19%)	
Bad	6 (17.14%)	6 (6.38%)	
Very bad	3 (8.57%)	2 (2.13%)	
**Nutrition Status**			.867
Healthy	10 (28.57%)	41 (43.61%)	
Unhealthy	14 (40.0%)	22 (23.40%)	
Undecided	11 (31.4%)	31 (32.9%)	
**Smoking**			.169
Smoker	12 (34.2%)	20 (21.2%)	
Non-smoker	23 (65.71%)	74 (78.73%)	
**Alcohol Drinking Status**			.508
Frequently drink alcohol	2 (5.74%)	8 (8.53%)	
Rarely drink alcohol	12 (34.2%)	23 (24.4%)	
Do not drink	21 (60.0%)	63 (67.02%)	
**Exercise Status**			.95
Exercise (+)	11 (31.4%)	29 (30.8%)	
Exercise (-)	24 (68.5%)	65 (69.1%)	
**Hobby Status**			.282
Hobby	6 (17.14%)	29 (30.85%)	
No hobby	24 (68.5%)	52 (55.31%)	
Uncertain	5 (14.28%)	13 (13.83%)	
**Anxiety Levels**			.015^*^
Minimum anxiety	4 (11.42%)	28 (29.78%)	
Mild anxiety	11 (31.42%)	32 (34.04%)	
Moderate anxiety	7 (20.0%)	21 (22.34%)	
Severe anxiety	13 (37.14%)	13 (13.83%)

**Table 3.** Chi-square test results between sex and related parameters.

Parameter	N	df	χ^2^	Cramer’s V	Effect Size	Raw p-value	Bon. Adjusted p-value	Significance
Grade	129	5	8.359	0.255	Medium	0.138	1.000	NS
Academic Achievement	129	2	0.895	0.083	Small	0.639	1.000	NS
Economic Status	129	4	4.258	0.182	Medium	0.372	1.000	NS
Peer Bullying	129	3	13.581	0.324	Large	0.004	0.052	*
Occupational Satisfaction	129	2	1.202	0.097	Small	0.548	1.000	NS
Earthquake Exposure Status	129	1	0	–	–	1.000	1.000	NS
Smoking	129	1	7	0.233	Medium	0.008	0.106	NS
Alcohol	129	2	4.79	0.193	Medium	0.091	1.000	NS
Exercise Status	129	1	1.012	0.089	Small	0.314	1.000	NS
Hobby Status	129	2	2.5	0.139	Medium	0.287	1.000	NS
Nutrition Status	129	2	4.907	0.195	Medium	0.086	1.000	NS
Academic Status	129	1	0.383	0.054	Small	0.536	1.000	NS
Anxiety Levels	129	3	6.686	0.228	Medium	0.083	1.000	NS

Notes: Bon = Bonferroniχ^2^ = chi-square statistic; Cramer’s V = effect size measure; * NS = not significant. Effect size benchmarks (k = 1): Small < 0.10.

Medium 0.10–0.30, Large ≥ 0.30.

*Peer bullying was significant only under FDR (p_FDR = .046), not under Bonferroni.

**Table 4.** Logistic regression with sex as a predictor.

Model Fit			
Statistic	Value		
Cox-Snell R^2^	0.3235		
Nagelkerke R^2^	0.4513		
McFadden R^2^	0.3097		
LR χ^2^ (30)	50.42, *p* = .011*		
AIC	174.38		
BIC	263.03		
Hosmer–Lemeshow χ^2^	4.55 (*p* = .805)		
**Significant Predictors (*p < .05*)** Only Exercise Status_2 reached significance in the multivariate model:			
Predictor	OR	95% CI	*p*
Exercise Status (Level 2 vs 1)	4.18	1.14–15.36	.031*
**Classification Performance**			
Metric	Value		
Overall Accuracy	83.7%		
Sensitivity	94.3%		
Specificity	61.9%		
AUC-ROC	0.844		
5-fold CV Accuracy	70.6% ± 6.7%

**Table 5.** Items, response options, and coding of the self-report questionnaire.

Construct	Response options	Coding in analysis
Academic Achievement	1 = Successful; 2 = Unsuccessful; 3 = Undecided	Categorical (3 levels)
Occupational Satisfaction	1 = Satisfied; 2 = Unsatisfied; 3 = Undecided	Categorical (3 levels)
Peer Bullying	1 = Never; 2 = Rarely; 3 = Sometimes; 4 = Often	Categorical (4 levels)
Economic Status	1 = Very good; 2 = Good; 3 = Normal; 4 = Bad; 5 = Very bad	Categorical (5 levels)
Smoking	1 = Yes; 0 = No	Binary (Yes/No)
Alcohol Use	1 = Frequently; 2 = Rarely; 3 = Do not drink	Categorical (3 levels)
Exercise Status	1 = Yes; 0 = No	Binary (Yes/No)
Nutrition Status	1 = Healthy; 2 = Unhealthy; 3 = Undecided	Categorical (3 levels)
Hobby Status	1 = Yes; 2 = No; 3 = Uncertain	Categorical (3 levels)
Academic Status (Pressure)	1 = Yes; 0 = No	Binary (Yes/No)
Earthquake Exposure Status	1 = Yes (affected through family); 0 = No direct family involvement	Binary (Yes/No)
Grade (year of study)	1 = 1st year … 6 = 6th year	Categorical (6 levels)
Anxiety (dichotomous)	1 = Yes; 2 = No; 3 = Undecided	Categorical (3 levels)

### Logistic regression model

All 13 categorical variables listed in Table 5 were entered simultaneously into a binary logistic regression model. No pre-filtering based on univariate significance was applied. Each categorical variable was dummy-coded with the first level (Level 1) serving as the reference category, resulting in a total of 30 predictor columns. The outcome variable (not shown in the mapping) was modelled as a binary event. Model fit was assessed using the Cox-Snell, Nagelkerke, and McFadden pseudo-R^2^ statistics, the likelihood ratio test, Akaike’s Information Criterion (AIC), Bayesian Information Criterion (BIC), and the Hosmer–Lemeshow goodness-of-fit test. Classification performance was evaluated through overall accuracy, sensitivity, specificity, and the area under the receiver operating characteristic curve (AUC-ROC). Five-fold cross-validation (CV) was performed to estimate out-of-sample predictive accuracy.

### Patient and public involvement

Patient and Public Involvement was not integrated into the study design, data collection, analysis, or interpretation. This is attributable to the specific focus and scope of the research, which aimed to explore sex-associated psychological and behavioral risk factors among medical students indirectly affected by the Kahramanmaras earthquake (i.e. via family members).

## Results

### Sex differences in psychosocial and behavioral parameters

A series of chi-square tests of independence was conducted to examine associations between sex (male/female) and each of the 13 categorical variables. Table 3 presents the full results, including chi-square values, degrees of freedom, *p*-values, and Cramer’s V effect sizes.

After applying a Bonferroni correction (adjusted α = 0.05/13 = 0.0038), none of the associations remained significant, because the raw *p*-value for peer bullying (p_raw = .004) exceeded the adjusted threshold (0.004 > 0.0038). Using the less conservative Benjamini–Hochberg false discovery rate (FDR) procedure (α = 0.05), peer bullying was significant (p_FDR = .046). All other variables were nonsignificant under both correction methods.

Raw associations approaching conventional significance (p_raw < .10) were observed for Smoking (χ^2^ (1) = 7.000, p_raw = .008, p_Bonferroni = .106, p_FDR = .053, V = 0.233), Anxiety Levels (χ^2^ (3) = 6.686, p_raw = .083, p_FDR = .237, V = 0.228), Nutrition Status (χ^2^ (2) = 4.907, p_raw = .086, p_FDR = .237, V = 0.195), and Alcohol (χ^2^ (2) = 4.790, p_raw = .091, p_FDR = .237, V = 0.193). None of these survived either correction. The remaining variables showed no meaningful uncorrected association with sex: Economic Status (χ^2^ (4) = 4.258, *p* = .372), Hobby Status (χ^2^ (2) = 2.500, *p* = .287), Occupational Satisfaction (χ^2^ (2) = 1.202, *p* = .548), Academic Achievement (χ^2^ (2) = 0.895, *p* = .639), and Earthquake Exposure Status (χ^2^ (1) = 0.000, *p* = 1.000).

### Comparison of participants affected and non-affected through family

Descriptive statistics comparing participants whose families were directly affected by the earthquake with those whose families were not affected are presented in Table 2.

No significant difference was found in the distribution of sex between the two groups (*p* = .522). The proportion of male and female participants was similar among those affected through family (31.4% male, 68.5% female) and those not affected (32.9% male, 67.02% female).

### Model fit and overall performance

The full logistic regression model was significant, likelihood ratio χ^2^ (30) = 50.42, *p* = .011. Pseudo‑R^2^ values indicated moderate explanatory power (Cox‑Snell R^2^ = 0.3235, Nagelkerke R^2^ = 0.4513, McFadden R^2^ = 0.3097). The model showed good calibration, with a non‑significant Hosmer–Lemeshow test (χ^2^ = 4.55, *p* = .805). The AIC was 174.38 and the BIC was 263.03. The full logistic regression model (Table 4) was significant, likelihood ratio χ^2^ (30) = 50.42, *p* = .011, with exercise status emerging as the sole significant predictor.

### Significant predictors

Among the predictors, only Exercise Status (Level 2 vs. Level 1) reached statistical significance (*p* <.05). The odds of the outcome were approximately 4.18 times higher for individuals at Exercise Status Level 2 compared to Level 1 (OR = 4.18, 95% CI = 1.14–15.36, *p* = .031).

### Classification accuracy

The model correctly classified 83.7% of cases (overall accuracy). Sensitivity was 94.3% and specificity was 61.9%. The AUC‑ROC was 0.844, indicating good discriminative ability. However, five‑fold cross‑validation yielded a lower mean accuracy of 70.6% (±6.7%), suggesting some degree of overfitting, which is expected given the number of predictors (30) relative to the sample size (*N* = 129). The Hosmer–Lemeshow test confirmed that the model’s predicted probabilities were well calibrated.

The distribution of anxiety severity categories by sex is presented in Table 6. Although a chi-square test on this 2 × 4 contingency table was non-significant (Table 3), female students appeared more frequently in the moderate and severe categories (25.3% and 24.1% vs. 14.3% and 11.9% in males).

**Table 6.** Anxiety category distribution by sex.

Anxiety Category (BAI)	Female *n* = 87	Male *n* = 42	Total *N* = 129
Minimum (0–7)	20 (23.0%)	12 (28.6%)	32 (24.8%)
Mild (8–15)	24 (27.6%)	19 (45.2%)	43 (33.3%)
Moderate (16–25)	22 (25.3%)	6 (14.3%)	28 (21.7%)
Severe (26–63)	21 (24.1%)	5 (11.9%)	26 (20.2%)
Total	87 (100%)	42 (100%)	129 (100%)

## Discussion

This study investigated sex-based disparities in psychosocial distress among 129 medical students indirectly exposed to the 2023 Kahramanmaras earthquakes. After applying a conservative Bonferroni correction for multiple comparisons, none of the initially observed sex differences remained statistically significant. However, the Benjamini–Hochberg false discovery rate (FDR) procedure (α = 0.05) identified peer bullying as significant (*p*_FDR = .046). This suggests that peer bullying may be a sex-related factor worth further investigation, but the evidence is not robust to the strictest correction.

It is worth noting that several variables, such as smoking (χ^2^ (1) = 7.000, *p*_raw = .008), showed nominally significant raw *p*-values but failed to remain significant after correction using either method. These effects, which are of moderate magnitude, might represent real but small population differences that a larger study could detect. The discrepancy between raw effect sizes and corrected *p* values is typical in small samples, where statistical power is limited. These effect sizes should not be dismissed outright; they may represent genuine population differences that a larger study could confirm.

The comparison between participants whose families were directly affected and those who were not (Table 2) provides a separate perspective. Students with affected family members reported lower occupational satisfaction and more severe anxiety – differences that were not part of the sex-specific chi-square tests and thus remain informative. This suggests that the proximity of the trauma (i.e. knowing a close family member was harmed) is a stronger driver of psychological distress than demographic variables such as sex. The absence of a sex difference in anxiety categories but a clear effect of family-exposure status reinforces the view that indirect exposure, through close social ties, can generate measurable distress.

The logistic regression model identified exercise status as the only significant predictor of sex (OR = 4.18), but this finding should be treated with caution.

Taken together, these results lead to a more circumscribed conclusion than the uncorrected analyses initially suggested. Sex may still play a role in shaping distress after indirect disaster exposure, but the evidence from this study is not strong. The only robust finding is a sex difference in peer bullying, which could be a pre-existing vulnerability factor rather than a direct earthquake effect. The large uncorrected effect sizes for other variables underscore the need for larger, adequately powered studies.

## Context of disaster and indirect exposure

The Kahramanmaras earthquakes produced severe psychological distress across directly exposed populations. For instance, a study of 947 adolescents who survived the earthquakes found that posttraumatic stress reactions explained 97.2% of their mental symptoms, and that their general mental symptom levels were at a pathological level [21].

The significance of anxiety in this sample remains important when viewed against the backdrop of the Kahramanmaras earthquakes. Even though these students were located ~1,000 km from the epicenter, they were connected to the disaster through family, friends, and the intense national media coverage that followed. The finding that those with directly affected family members had more severe anxiety supports the conservation of resources theory: the threat of resource loss (family safety) can initiate a stress response even when the individual is physically safe [5].

## Conclusion

After rigorous correction for multiple testing, no sex differences survived the most conservative (Bonferroni) adjustment. Using a less conservative false discovery rate approach, peer bullying was associated with sex (*p*_FDR = .046). These results raise the possibility of subtle sex-specific patterns, but larger studies are needed for confirmation.

## Suggestions for further studies

Longitudinal designs are essential to track the trajectory of distress over time and to disentangle acute reactions from chronic psychopathology. Larger cohorts that include both directly and indirectly exposed groups, with a wider range of psychosocial measures and validated instruments, would permit more definitive conclusions about the role of sex. Qualitative approaches could also illuminate the mechanisms underlying the sex-specific patterns hinted at here, particularly the peer bullying finding.

## Limitations

Low sample size is one of the main limitations of our study. Furthermore, relying on self-reported scales may not provide a clear diagnosis of anxiety. In addition to the low sample size noted previously, the study is subject to substantial self-selection bias. Participants were recruited via convenience sampling through social media platforms (Facebook and WhatsApp groups). Because the recruitment posts reached an unknown number of potential viewers, a response rate cannot be calculated, and the proportion of the eligible population represented by our sample of 129 students is unknown. It is possible that medical students who chose to participate differed systematically from those who did not – for example, those with higher levels of distress, greater health awareness, or more free time may have been overrepresented.

**Figure 2.**
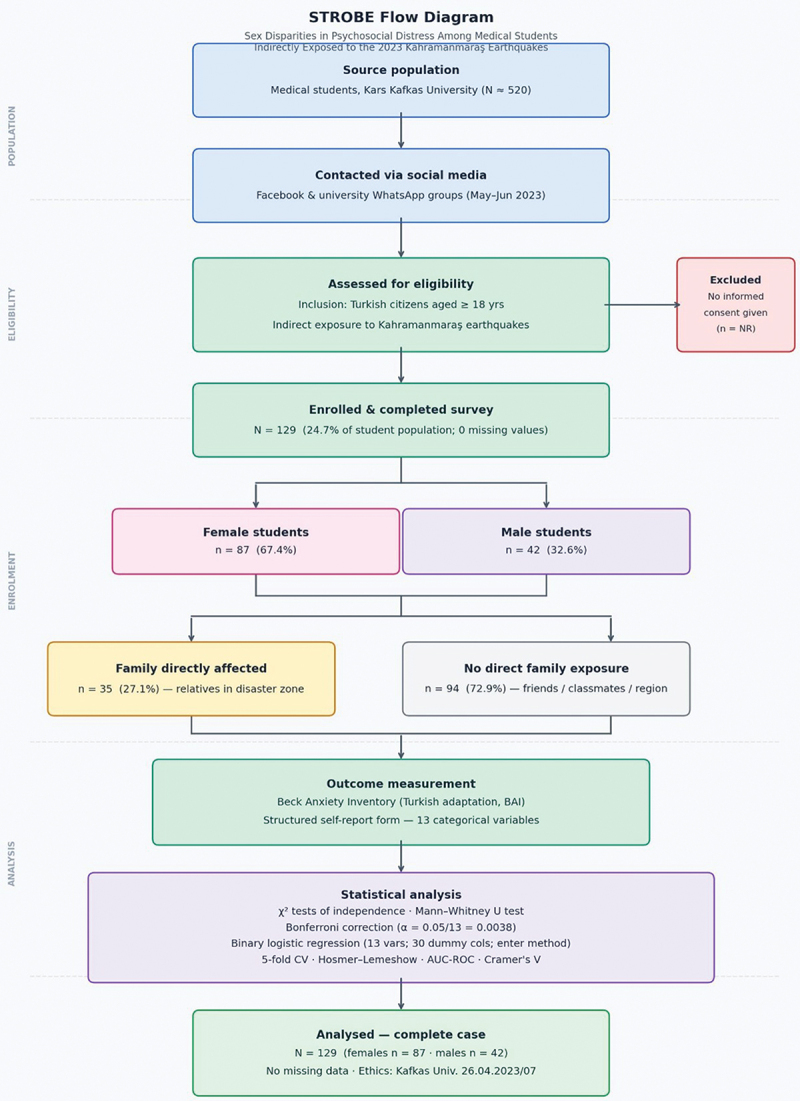
Flow diagram. The diagram (Figure 2) illustrates the participant flow from the source population through eligibility screening, enrolment, subgroup stratification, and final analysis, in accordance with the Strengthening the Reporting of Observational Studies in Epidemiology guidelines for cross-sectional studies.

## Supplementary Material

STROBE.doc

## Data Availability

The datasets generated and analyzed during the current study are not publicly available due to privacy and ethical restrictions protecting participant confidentiality, given the sensitive nature of mental health data collected from a potentially vulnerable population in a post-disaster context. Anonymized data may be available from the corresponding author upon reasonable request and with appropriate data sharing agreements consistent with ethical approvals.
