# Supplementary material for: Sex disparities in psychosocial distress among medical students with indirect exposure to the 2023 Kahramanmaras earthquakes
Source: Glob Health Action. 2026 Jul 13;19(1):2698916. doi: 10.1080/16549716.2026.2698916 (PMC13366645; doi:10.1080/16549716.2026.2698916)
Supplement: STROBE.doc [file ZGHA_A_2698916_SM7412.doc]

**STROBE Statement Checklist**

1. **Gender Disparities in Psychosocial Distress Among Medical Students with Indirect Exposure to the 2023 Kahramanmaras Earthquakes**

| **Item No** | **Recommendation** | **Location in Manuscript** | **Assessment** |
| --- | --- | --- | --- |
| **Title and abstract** |  |  |  |
| 1 | (a) Indicate the study's design with a commonly used term in the title or the abstract | **Title**: Does not explicitly state design **Abstract**: "In this cross-sectional study" (line 6) | **Partially Met** – Design stated in abstract but not in title |
|  | (b) Provide in the abstract an informative and balanced summary of what was done and what was found | Abstract (lines 1-20) includes background, methods, results, and conclusion | **Yes** |
| **Introduction** |  |  |  |
| 2 | Explain the scientific background and rationale for the investigation being reported | Introduction (Section 1, paragraphs 1-5) provides context on earthquake impact, gender vulnerability, and research gap | **Yes** |
| 3 | State specific objectives, including any prespecified hypotheses | End of Introduction (paragraphs 6-7): Two specific objectives stated; no explicit hypotheses | **Partially Met** – Objectives clear, hypotheses not prespecified |
| **Methods** |  |  |  |
| 4 | Present key elements of study design early in the paper | Section 2.1 (Participants): "cross-sectional study" stated; design elements described | **Yes** |
| 5 | Describe the setting, locations, and relevant dates, including periods of recruitment, exposure, follow-up, and data collection | Section 2.1: "Data collection commenced approximately three months after the earthquake (May 2023)"; recruitment via social media; Turkey | **Yes** |
| 6 | (a) Give the eligibility criteria, and the sources and methods of selection of participants | Section 2.1: "Turkish medical students aged 18 years or older... indirectly affected through their families"; recruited via Facebook/WhatsApp groups | **Yes** |
|  | (b) For matched studies – not applicable | N/A | N/A |
| 7 | Clearly define all outcomes, exposures, predictors, potential confounders, and effect modifiers. Give diagnostic criteria, if applicable | Outcomes (anxiety, academic stress, behaviors) defined in Tables 2-3; exposure (family affected) defined; no diagnostic criteria provided for anxiety (self-reported) | **Partially Met** – Outcomes defined but no validated diagnostic criteria |
| 8 | For each variable of interest, give sources of data and details of methods of assessment. Describe comparability of assessment methods | Section 2.2 mentions survey; specific survey instrument not described; no validation details | **No** – Survey instrument not described |
| 9 | Describe any efforts to address potential sources of bias | Section 2.2 mentions verifying Chi-square assumptions; no other bias mitigation described | **Partially Met** – Limited bias discussion |
| 10 | Explain how the study size was arrived at | No sample size calculation or justification provided | **No** |
| 11 | Explain how quantitative variables were handled in the analyses. If applicable, describe which groupings were chosen and why | Section 2.2: Chi-square for categorical variables; Mann-Whitney U for non-normal continuous variables; groupings shown in Tables 2-3 | **Yes** |
| 12 | (a) Describe all statistical methods, including those used to control for confounding | Section 2.2: Chi-square tests, Cramer's V, Mann-Whitney U; no adjustment for confounders | **Partially Met** – Methods described but no confounding control |
|  | (b) Describe any methods used to examine subgroups and interactions | Gender as primary subgroup; Table 3 shows gender-based analyses | **Yes** |
|  | (c) Explain how missing data were addressed | Section 2.2: "No missing values were identified" | **Yes** |
|  | (d) Cohort study – N/A | N/A | N/A |
|  | (e) Describe any sensitivity analyses | No sensitivity analyses reported | **No** |
| **Results** |  |  |  |
| 13 | (a) Report numbers of individuals at each stage of study | Section 2.1: 129 participants (87 female, 42 male) | **Yes** |
|  | (b) Give reasons for non-participation | Section 2.1: "Participants who did not provide informed consent were unable to complete the study" | **Yes** |
|  | (c) Consider use of a flow diagram | No flow diagram provided | **No** |
| 14 | (a) Give characteristics of study participants | Table 1 (literature review) and Table 2 (participant characteristics by exposure status); gender distribution, academic, economic status | **Yes** |
|  | (b) Indicate number of participants with missing data for each variable | Section 2.2: "No missing values were identified" | **Yes** |
|  | (c) Cohort study – N/A | N/A | N/A |
| 15 | Cross-sectional study – Report numbers of outcome events or summary measures | Tables 2 and 3 present counts, percentages, and statistical test results for all outcomes | **Yes** |
| 16 | (a) Give unadjusted estimates and, if applicable, confounder-adjusted estimates | Table 3: Unadjusted Chi-square results with Cramer's V; no adjusted estimates | **Partially Met** |
|  | (b) Report category boundaries when continuous variables were categorized | All variables categorical; boundaries shown in tables | **Yes** |
|  | (c) If relevant, consider translating estimates of relative risk into absolute risk – Not applicable | N/A | N/A |
| 17 | Report other analyses done – e.g., subgroup, sensitivity | Gender subgroup analysis (Table 3) reported; no sensitivity analyses | **Partially Met** |
| **Discussion** |  |  |  |
| 18 | Summarise key results with reference to study objectives | Section 4 (Discussion), paragraphs 1-2 summarize gender differences and indirect exposure findings | **Yes** |
| 19 | Discuss limitations of the study, taking into account sources of potential bias or imprecision | Section "Limitations" (after Discussion): low sample size, self-reported scales noted | **Yes** – Brief but present |
| 20 | Give a cautious overall interpretation of results considering objectives, limitations, multiplicity of analyses, results from similar studies, and other relevant evidence | Section 4 discusses findings in context of literature; acknowledges limitations; interpretations appropriately cautious | **Yes** |
| 21 | Discuss the generalisability (external validity) of the study results | Section 4: Results specific to medical students; indirectly exposed population; not generalized broadly | **Partially Met** – Limited discussion of generalizability |
| **Other information** |  |  |  |
| 22 | Give the source of funding and the role of the funders | Funding section: "None"; role of funders not applicable | **Yes** |
